# Supplementary material for: Conductance through a helical state in an Indium antimonide nanowire
Source: Nat Commun. 2017 Sep 7;8:478. doi: 10.1038/s41467-017-00315-y (PMC5589903; doi:10.1038/s41467-017-00315-y)
Supplement: Supplementary file 1 — Supplementary Information [file 41467_2017_315_MOESM1_ESM.pdf]

File name: Supplementary Information
Description: Supplementary Figures, Supplementary Notes and Supplementary References.

File name: Peer Review File
Description:

SUPPLEMENTARY NOTE 1: NUMERICAL SIMULATIONS OF THE CONDUCTANCE THROUGH A HELICAL STATES

Poisson calculations in a 3D nanowire device

Observing the helical gap in a semiconducting nanowire crucially depends on the smoothness of the electrostatic potential profile between the two contacts [1]. When the potential profile changes too abruptly, it forms a tunnel barrier which suppresses conductance well below quantized values, thereby masking features of the helical gap. On the other hand, if the potential varies on a length scale much larger than the characteristic spin-orbit coupling length l_{SO}, transmission through the 'internal state' (the smaller-momentum state of the two right-moving states in the bottom of the lower band) is suppressed. This reduces the first $2e^2/h$ plateau in the conductance to a $1e^2/h$ plateau, thereby concealing again the helical gap. Because of the crucial role of the electrostatic potential, we perform realistic Poisson calculations to compute the potential $\phi(\vec{r})$ in the nanowire (with $\vec{r} = (x, y, z)$), solving the Poisson equation of the general form

$$\nabla^2 \phi(\vec{r}) = -\frac{\rho(\vec{r})}{\epsilon}, \tag{1}$$

with ϵ the dielectric permittivity and ρ the charge density. For the charge density ρ, we apply the Thomas-Fermi approximation [2]

$$\rho(\vec{r}) = \frac{e}{3\pi^2\epsilon}\left(\frac{2m^* e\phi(\vec{r})}{\hbar^2}\right)^{3/2}, \tag{2}$$

where m^* is the effective mass of InSb.
For a given charge density ρ, we solve Supplementary Eq. 1 numerically for the potential using the finite element package FEniCS [3]. We model the two normal contacts as metals with a fixed potential $V_{\mathrm{N}} = 0.22$ V, assuming a small work function difference between the nanowire and the normal contacts. The back gate is modeled as a fixed potential V_{G} along the bottom surface of the dielectric layer. We use the dielectric permittivities for InSb and SiN in the wire and the dielectric layer respectively. The FEM mesh, with its dimensions and boundary conditions, is depicted in Supplementary Fig. 1a.
We apply the Anderson mixing scheme [4] to solve the nonlinear equation formed by Supplementary Eqs. 1 and 2 self-consistently. An example of a self-consistent Poisson potential with Thomas-Fermi density is plotted in Supplementary Fig. 1b.

Conductance calculations in a 1D model with a projected potential barrier

To apply the 3D Poisson potential in a simple 1D nanowire model, we convert the three-dimensional potential $\phi(x, y, z)$ to a one-dimensional effective potential barrier $\hat{\phi}(x)$ by projecting ϕ on the transverse wave functions $\psi(y, z)$ in the nanowire:

$$\hat{\phi}(x) = \langle \psi(y, z)|\phi(x, y, z)|\psi(y, z)\rangle. \tag{3}$$

To do this, we compute the eigenenergies of the Hamiltonian of a two-dimensional cross section at a point x_0 along the wire, with a corresponding potential $\phi(x_0, y, z)$. The effective potential barrier is then given by the ground state of the Hamiltonian. The longitudinal variation of the potential barrier is obtained by computing the ground state of the transverse Hamiltonian at many points along the wire. An example of the projected potential is given in Supplementary Fig. 1c with the solid-black curve. Due to rough boundary conditions in the FEM mesh (see the edges of the dielectric layer and the normal contacts in the potential of Supplementary Fig. 1b), the projected potential $\hat{\phi}(x)$ shows some roughness that may cause unwanted scattering events (see black curve in Supplementary Fig. 1c). To avoid this, we fit $\hat{\phi}(x)$ to a linear combination of hyperbolic tangents, given by

$$V(x) = \frac{E_{\mathrm{a}}}{2}\left[\tanh\left(\frac{x - x_{\mathrm{s}} + W/2}{\lambda/2}\right) - \tanh\left(\frac{x - x_{\mathrm{s}} - W/2}{\lambda/2}\right)\right] + E_{\mathrm{s}}. \tag{4}$$

Here, E_{a} is the amplitude, W the width and E_{s} the downshift in energy of the potential barrier, which varies along x on a typical length scale λ, as indicated in Supplementary Fig. 1c. The horizontal shift of the barrier to the middle of the nanowire is denoted by $x_{\mathrm{s}} = 500$ nm. The parameter λ expresses the smoothness of the barrier. We find that λ

is close to zero when no charge is present in the wire and the boundary conditions result in an abrupt step in the potential between the contacts and the uncovered part of the wire. When charge enters the wire, it screens the electric field, thereby smoothening the potential. For a QPC length of 325 nm we find in this regime $\lambda \approx 80$ nm. The value of λ is reduced for smaller QPC lengths, but saturates to $\lambda \approx 80$ nm for longer QPC lengths. Moreover we find that λ has only a little dependency on the back gate voltage $V_{\rm G}$ or the applied magnetic field B (Supplementary Fig. 1d). Taking advantage of the latter and the fact that we are interested in the conductance of the wire in the vicinity of the helical-gap feature – where the screening *is* present – we assume λ constant in $V_{\rm G}, B$ space for the conductance calculation. For the conductance calculations we consider transport through a two-mode nanowire described by the Hamiltonian

$$\mathcal{H} = \left[\frac{\hbar^2 k_x^2}{2m^*} + V(x)\right]\sigma_0 + \alpha\sigma_y k_x + \frac{1}{2} g\mu_{\rm B} B(\sigma_{\rm x}\sin\theta + \sigma_{\rm y}\cos\theta), \tag{5}$$

where σ denote the Pauli matrices (with σ_0 the identity matrix) and $V(x)$ is fit to the projected potential barrier, as expressed in Supplementary Eqs. 3 and 4. Spin-orbit coupling strength is given by $\alpha = \hbar^2/m^* l_{\rm SO}$ where $l_{\rm SO}$ we use as a free parameter. We take the effective mass $m^* = 0.014 m_0$ of InSb and $g = -38$ (unless stated otherwise) as estimated in the main text. Note that for the coordinate system used here, the wire lies along the x direction and θ is the angle between $B_{\rm SO}$ and the external magnetic field. The Hamiltonian Supplementary Eq. 5 is discretized on a mesh with lattice spacing $\Delta x = 4$ nm. Assuming translational invariance of the boundary conditions at the ends of the wire one arrives at the scattering problem that is solved using the Kwant package [5] to obtain the linear-response conductance within the Landauer-Büttiker formalism.

SUPPLEMENTARY NOTE 2: ANGLE-DEPENDENCE OF CONDUCTANCE IN RASHBA NANOWIRES

Theoretical model

We consider a one-dimensional nanowire with Rashba spin-orbit interaction (SOI) in an external magnetic field $\mathbf{B}$. The field $\mathbf{B}$ is oriented at an angle θ with respect to the effective magnetic field $\mathbf{B}_{\rm SO}$ due to Rashba SOI, as shown in Supplementary Fig. 1e. This setup is described by the Hamiltonian:[6]

$$H = \frac{p^2}{2m^*} + \frac{\alpha}{\hbar} p\sigma_{\rm y} + \frac{1}{2} E_{\rm Z}\left(\sin(\theta)\sigma_{\rm x} + \cos(\theta)\sigma_{\rm y}\right) . \tag{6}$$

In this expression, p is the momentum operator, m^* is the effective mass, α the Rashba SOI-strength, and $\sigma_{\rm x,y}$ the Pauli matrices. The Zeeman energy $E_{\rm Z} = g\mu_{\rm B} B$, where g is the g-factor, and $\mu_{\rm B}$ the Bohr magneton. In Supplementary Eq. (6) we assumed without loss of generality a magnetic field in the $x-y$-plane; the band structure however only depends on the relative angle θ of $\mathbf{B}$ with $\mathbf{B}_{\rm SO}$. The Rashba SO-strength α can be related to an effective length scale, the spin-orbit length

$$l_{\rm SO} = \frac{\hbar^2}{m\alpha} \tag{7}$$

and to an energy scale, the spin-orbit energy

$$E_{\rm SO} = \frac{m\alpha^2}{2\hbar^2} . \tag{8}$$

Defining length in units of $l_{\rm SO}$ and energy in units of $E_{\rm SO}$ it is possible to write the Hamiltonian in a convenient dimensionless form:

$$H = \frac{d^2}{dx^2} + 2\frac{d}{dx}\sigma_{\rm y} + \frac{1}{2}\frac{E_{\rm Z}}{E_{\rm SO}}\left(\sin(\theta)\sigma_{\rm x} + \cos(\theta)\sigma_{\rm y}\right) . \tag{9}$$

Proper units will be restored in the final result. In an translationally invariant nanowire, the wave vector k is a good quantum number and the Rashba Hamiltonian is readily diagonalized as [6]

$$E_{\pm}(k) = k^2 \pm \frac{1}{2}\sqrt{\left(\frac{E_{\rm Z}}{E_{\rm SO}}\right)^2 + 16k^2 + 8\frac{E_{\rm Z}}{E_{\rm SO}} k\cos(\theta)} . \tag{10}$$

The resulting band structure for a general angle θ is shown schematically in the left panel of Supplementary Fig. 1f. The band structure can be related to an idealized quantum point contact (QPC) conductance by counting the number of propagating modes at a given energy E (see right panel of Supplementary Fig. 1f). In the following we will derive from the band structure: (i) the size of the $1e^2/h$ plateaus in energy (denoted by $\Delta E_{\mathrm{Z},1}$ and $\Delta E_{\mathrm{Z},2}$). This is directly measurable using the finite bias dependence of the QPC conductance (measuring so-called QPC diamonds). (ii) The critical field for which the spin-orbit induced $2e^2/h$ conductance (the size of this plateau is denoted as ΔE_{SO}) vanishes. This allows for an estimate of the spin-orbit strength from the magnetic field dependence in experiment.

Size of Zeeman-induced gaps

In order to compute the size of the different QPC plateaus in energies, we need to compute the value of the minima and maxima of the bands $E_\pm(k)$. This can be done exactly using a computer algebra program (we used Mathematica), as it only involves solving for the roots of polynomials up to fourth order. The resulting expressions are however quite cumbersome, and it is more useful to find an approximate expression doing a Taylor approximation. Up to second order in $E_\mathrm{Z}/E_\mathrm{SO}$ we then find the simple expressions

$$\Delta E_{\mathrm{Z},1} \approx E_\mathrm{Z} \sin\theta\,, \tag{11}$$

$$\Delta E_{\mathrm{Z},2} \approx E_\mathrm{Z} \cos\theta\,. \tag{12}$$

Critical magnetic field for the spin-orbit induced $2e^2/h$-plateau

The spin-orbit induced $2e^2/h$ region persists only up to a critical Zeeman splitting $E_{\mathrm{Z,crit}}$, after which the two $1e^2/h$-plateaus merge into one. In the band structure, this corresponds to a transition from three extrema in $E_-(k)$ (two minima, one maximum) to only one minimum. The critical Zeeman splitting where this happens can be solved for exactly using Mathematica:

$$\frac{E_{\mathrm{Z,crit}}}{E_\mathrm{SO}} = \sqrt{\frac{54\cos(8\theta) + 3M_1^{\frac{2}{3}} + 6\left(3M_1^{\frac{1}{3}} - 4\right)\cos(4\theta) - 2M_1^{\frac{1}{3}} - 30}{M_2^{\frac{1}{3}}}} \tag{13}$$

where

$$M_1 = 68 - 86\cos(4\theta) - 36\cos(8\theta) + 54\cos(12\theta) + 512\sqrt{\sin^4(2\theta)\cos^2(2\theta)} \tag{14}$$

$$M_2 = 68 - 86\cos(4\theta) - 36\cos(8\theta) + 54\cos(12\theta) + 256\sqrt{\sin^2(2\theta)\sin^2(4\theta)} \tag{15}$$

For $\theta = 17^\circ$ this gives $E_{\mathrm{Z,crit}} = 2.386E_\mathrm{SO}$ and for $\theta = 10^\circ$ $E_{\mathrm{Z,crit}} = 2.695E_\mathrm{SO}$. When the value of the nanowire g-factor is extracted from experiment, the critical Zeeman splitting can be translated into a critical magnetic field. The magnetic field up to which the spin-orbit induced $2e^2/h$-plateau is still visible in experiment can then be used to set a *lower bound* on the spin-orbit energy. It is a lower bound, as for a given QPC potential the $2e^2/h$ may not be visible any more despite in principle being present in the band structure. A more detailed transport calculation can be used to improve on this bound.

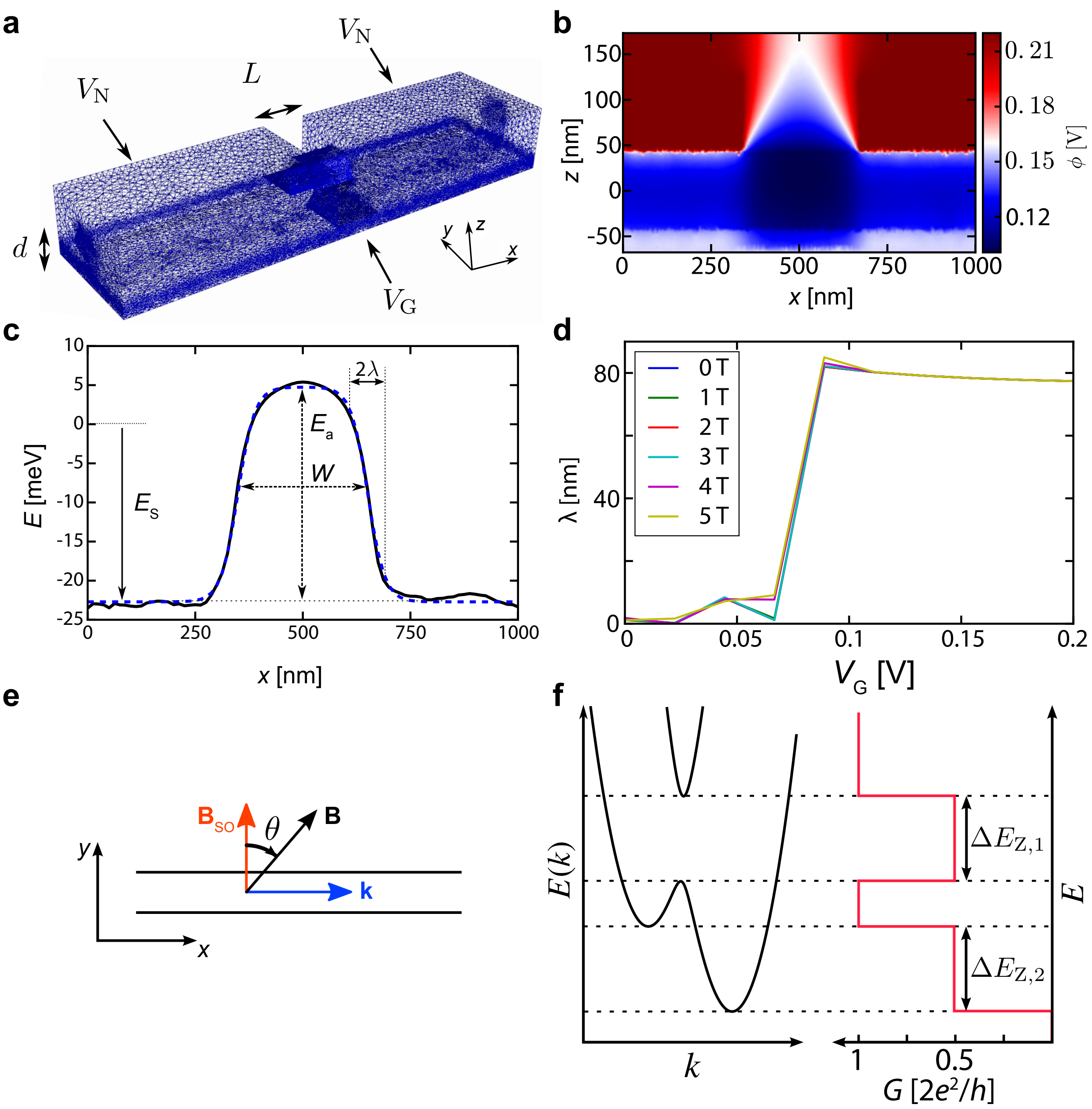

Supplementary Figure 1: Numerical model. a, Example of a finite element mesh used for 3D Poisson calculations. L denotes the QPC length (spacing between the two contacts), d the thickness of the dielectric layer, which is set to 20 nm. L is set to 325 nm for the simulations in the main text, and varied from 175 to 425 nm to show the length dependence of the helical gap feature in Supplementary Fig. 8. The two boundary conditions applied are a potential V_{N} on the contacts and a potential V_{G} underneath the dielectric layer. The mesh between the two contacts is left out for visibility purposes. **b,** Cross section plot of the 3D Poisson potential for $V_{\mathrm{G}} = 0.156\,\mathrm{V}$ and $V_{\mathrm{N}} = 0.22\,\mathrm{V}$. The cross section is taken along the wire axis (x-axis) for fixed $y = 0\,\mathrm{nm}$ in the middle of the wire. The effective QPC length runs from ~ 340 to $\sim 660\,\mathrm{nm}$. The nanowire is situated between $z = -50\,\mathrm{nm}$ and $z = 50\,\mathrm{nm}$. **c,** Projected potential $\hat{\phi}(x)$ (black curve) and fitted potential $V(x)$ (blue dashed curve) for $V_{\mathrm{G}} = 0.156\,\mathrm{V}$, corresponding to the potential of Supplementary Fig. 1b. Indicated are the fitting parameters E_{s}, E_{a}, W and λ of the function Supplementary Eq. 4. **d,** The fitting parameter λ as a function of back gate voltage V_{G}. Different colors denote different magnetic field strengths B. A jump in $\lambda \approx 0$ (abrupt step potential) to $\lambda \approx 80\,\mathrm{nm}$ occurs when charge enters the wire, screening the electric field. **e,** Rashba nanowire in an external magnetic field: the one-dimensional nanowire is oriented along the x-axis, and the spin-orbit field $\mathbf{B}_{\mathrm{so}}$ perpendicular, along the y-axis. The external magnetic field $\mathbf{B}$ forms an angle θ with respect to $\mathbf{B}_{\mathrm{so}}$. **f,** Schematic picture of the band structure $E(k)$ of a Rashba nanowire in a magnetic field (left panel) and the corresponding quantum point conductance G (right panel).

I. DEVICE 1 - ADDITIONAL DATA

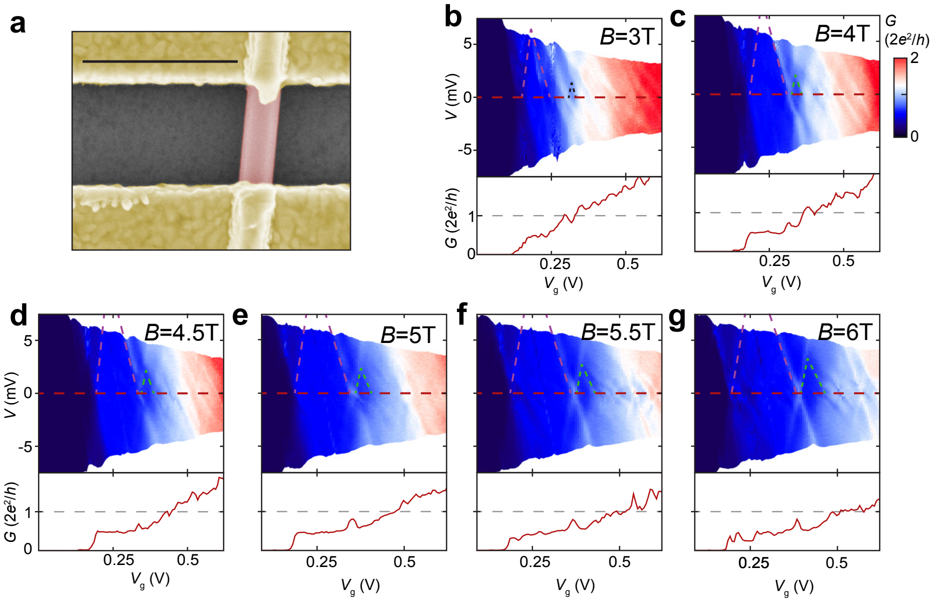

Supplementary Figure 2: Voltage bias spectroscopy of device 1. a, False color SEM image of device 1. The InSb nanowire is shown in red and Cr/Au contacts in yellow, scalebar corresponds to 500 nm. **b-g,** Conductance measurements as a function of QPC gate voltage V_g and source-drain bias voltage V at increasing magnetic field. Dotted lines indicate the helical gap as well as the $0.5 \cdot G_0$ plateau. The helical gap shows as feature stable in V and evolves linearly with magnetic field.

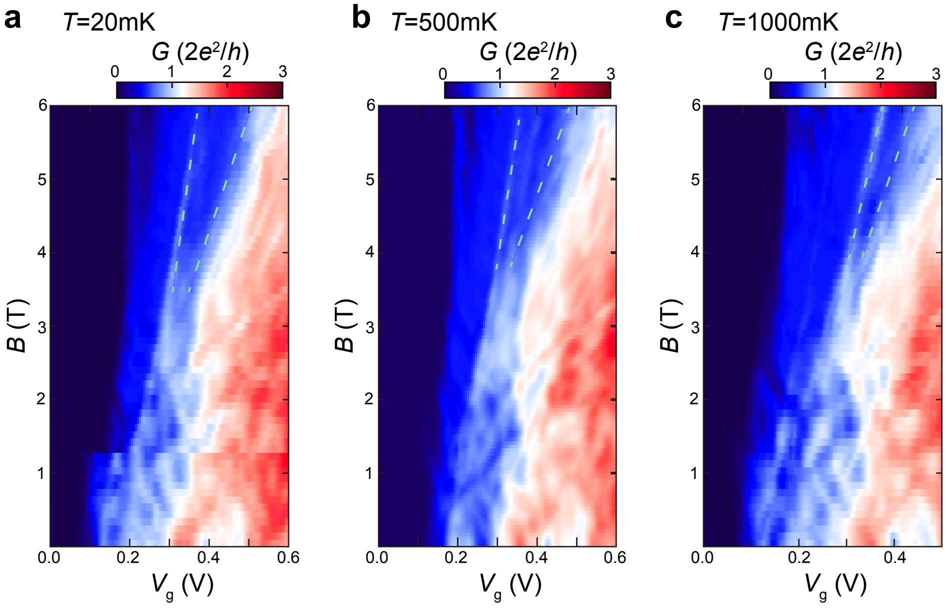

Supplementary Figure 3: Temperature dependence of the helical gap. Measurements of the differential conductance dI/dV ($V_{\mathrm{sd}} = 0\,\mathrm{mV}$) as function of magnetic field at **a,** $T = 20\,\mathrm{mK}$ **b,** $T = 500\,\mathrm{mK}$ **c,** $T = 1000\,\mathrm{mK}$. The helical gap (dotted lines) evolves similarly in all three measurements showing that it stays stable at increased temperatures as expected for the energy scale extracted for E_{SO}

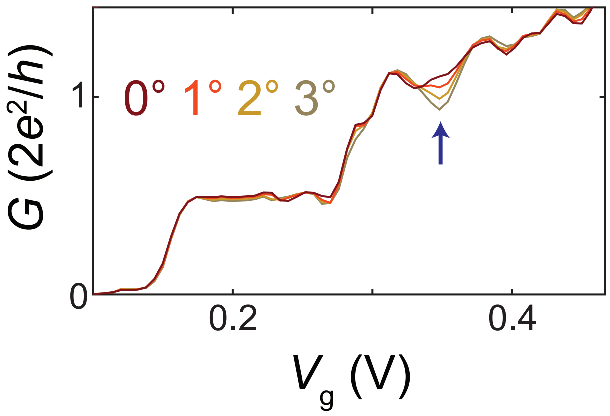

Supplementary Figure 4: Additional linecuts of the angle dependence. A small remaining conductance dip can be seen in the linecut at 0° in Fig. 4e. This is likely due to imperfect alignment between the external magnetic field and B_{SO}, either due to the limited range of the vector magnet or due to a small misalignment between the sample plane and the magnets x-y plane. Linecuts of Fig. 4e taken at 0°, 1°, 2° and 3° additionally show the strong angle dependence of the observed reentrance feature.

II. DEVICE 2 - DATA

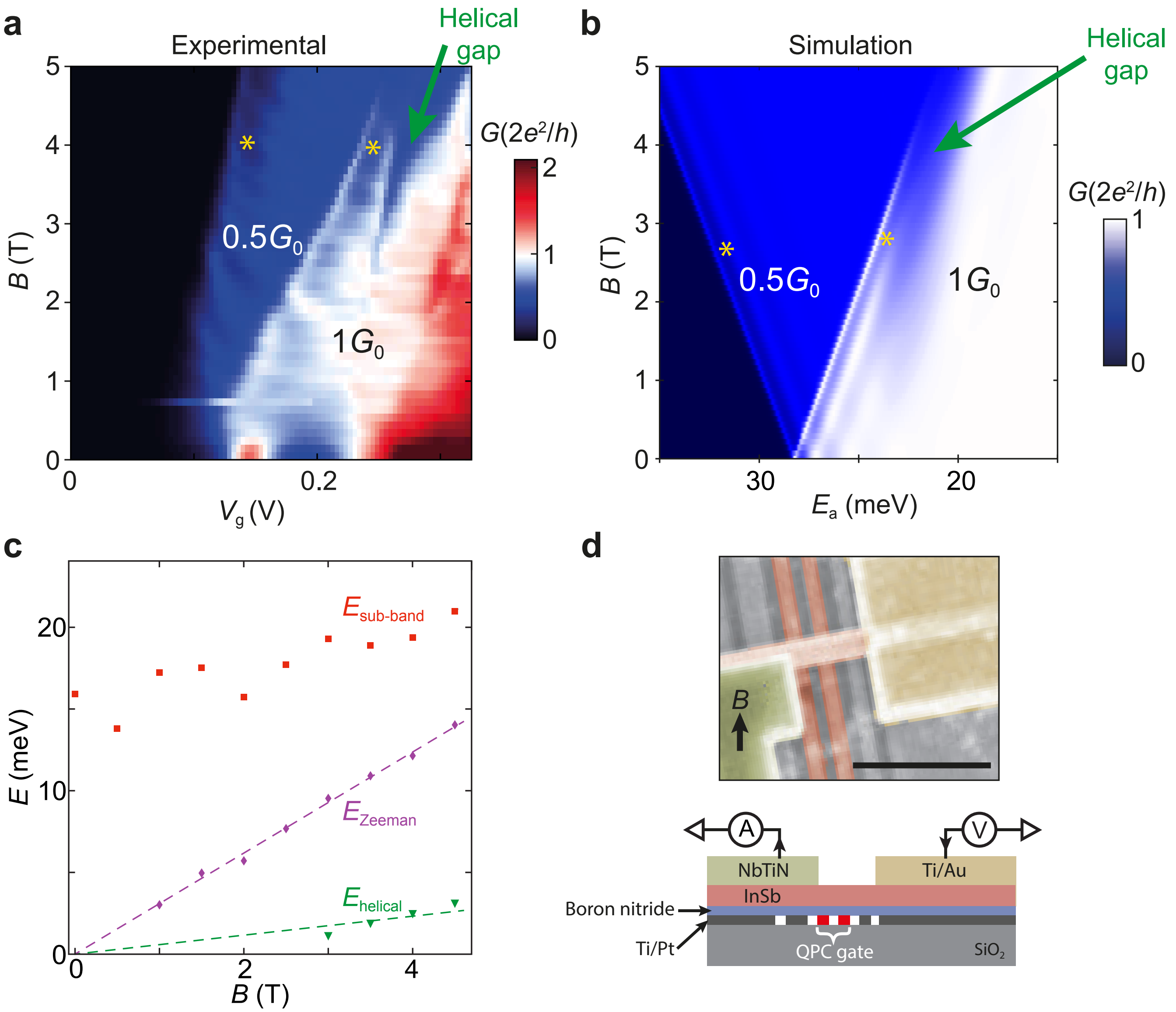

Supplementary Figure 5: Measurements of a second device. **a,** Differential conductance dI/dV as function of QPC gate Voltage V_g and magnetic field B. Around $B = 2\,\mathrm{T}$ a gap opens in the $1 \cdot G_0$ plateau and increases linearly with magnetic field. At the onset of the $0.5 \cdot G_0$ and the $1 \cdot G_0$ plateaus Fabry-Perot resonances are visible (yellow asterisk). In contrast to the helical gap the width of the resonances stays constant at changing magnetic field. **b,** Numerical simulations of the helical gap with $\theta = 10°$, $g = 53$ and $E_{SO} = 5.6\,\mathrm{meV}$. We use the potential parametrization as for the device discussed in the main text and find a good agreement with the data shown in **a** for $\lambda = 40\,\mathrm{nm}$ and $W = 300\,\mathrm{nm}$. **c,** Evolution of the energy levels with magnetic field extracted from the scans shown in Supplementary Fig. 6. Dotted lines show fits with intercept fixed at zero and we find a sub-band spacing $E_{\mathrm{subband}} = 18 \pm 2\,\mathrm{meV}$ (red) and g-factor $g = 53 \pm 1$. By comparing the slopes of $E_{\mathrm{Zeeman}} \sim E_Z \cos\theta$ (purple) and $E_{\mathrm{helical}} \sim E_Z \sin\theta$ (green) we find $\theta = 10° \pm 2°$. **d,** Cross section and false color SEM image of device 2. An InSb nanowire (orange) is contacted by one Ti/Au electrode (yellow) and one NbTiN electrode (green). Two bottom gates (red) are combined to form the QPC constriction. The black arrow indicates the orientation of the applied magnetic field. Measurements are taken at 20 mK with the use of standard lock-in technique (100 µV excitation at 73 Hz). Scalebar corresponds to 500 nm.

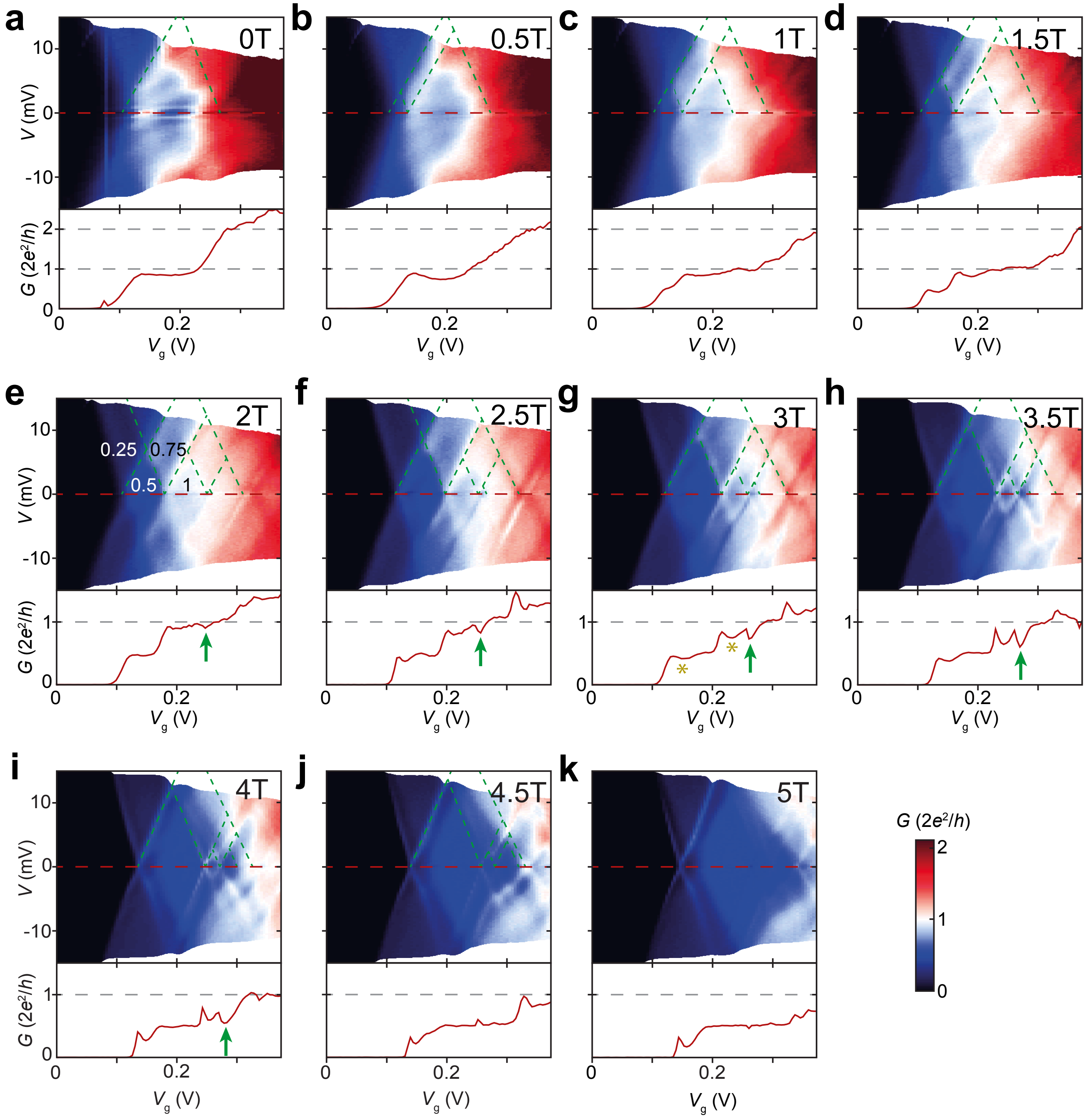

Supplementary Figure 6: Voltage bias spectroscopy at increasing magnetic fields of device 2. a-k, (Top) Differential conductance dI/dV as a function of QPC gate voltage V_g and bias voltage V. Conductance plateaus show up as diamond shaped region indicated by dashed green lines and can be used to extract the energy spacings shown in Supplementary Fig. 5c. Conductance traces in the bottom panels show line cuts taken at $V_{sd} = 0\,mV$. Green arrows in **e-i** indicate the position of the helical dip. Yellow asterisks in **g** indicate conductance dips originating from Fabry-Perot resonances also visible in Supplementary Fig. 5a. Numbers in **e** denote conductance in units of $2e^2/h$.

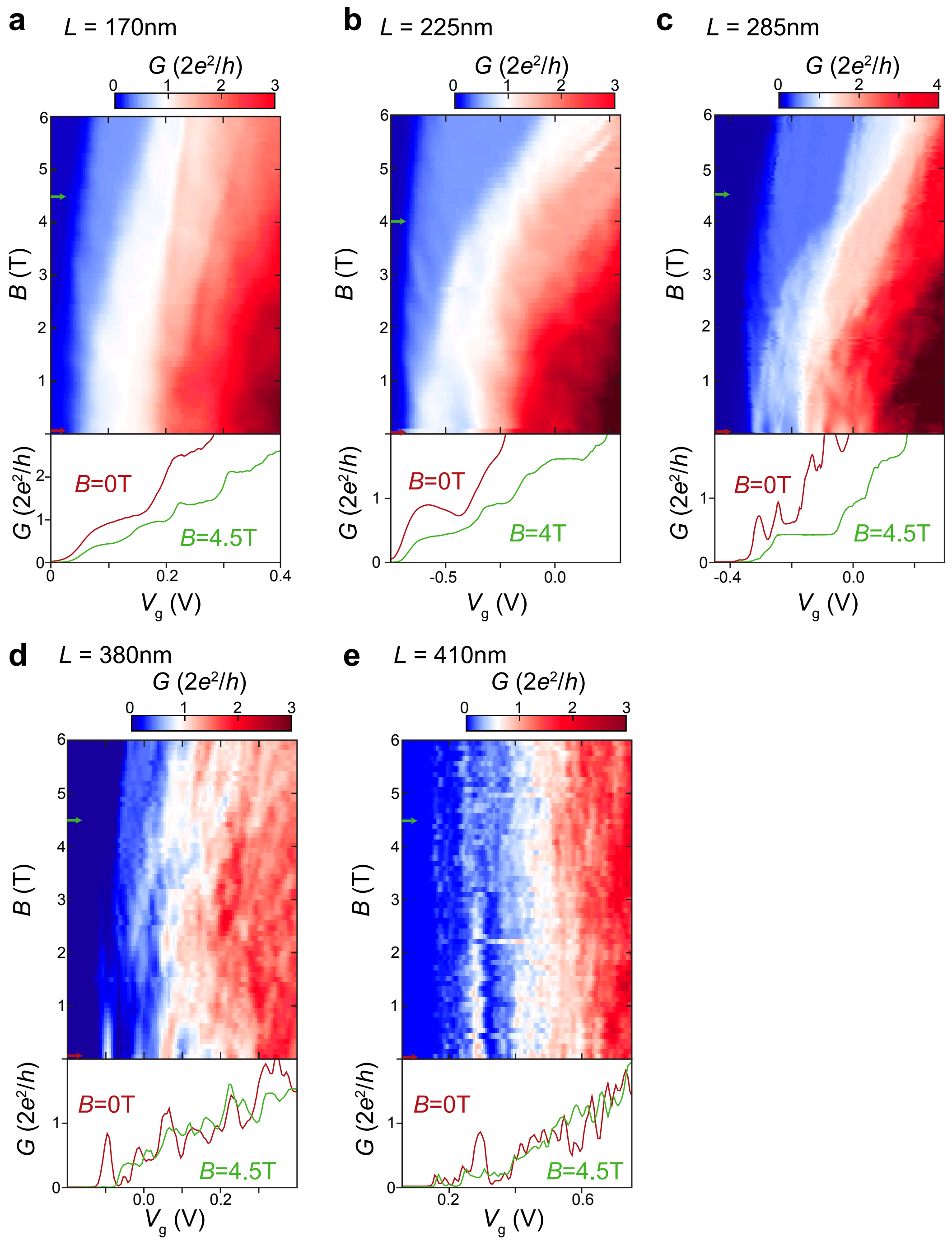

Supplementary Figure 7: Length dependence of nanowire QPCs. Magneto-conductance measurements (at $V_{sd} = 0\,\mathrm{mV}$) of QPCs with increasing length. The contact spacing L is changed in Steps of $\sim 50\,\mathrm{nm}$. **a-c,** are shorter and **d,e,** longer than device 1 ($L = 325\,\mathrm{nm}$). Line traces at 0 T and finite field are added in the bottom panel. The short channel devices **a,b,**show well defined and flat plateaus throughout the full magnetic field range. For intermediate channel lengths (**c**) resonances start to appear that modify the conductance at low magnetic fields. Long channel devices **d,e,** are dominated by backscattering and conductance fluctuations throughout the full magnetic field range.

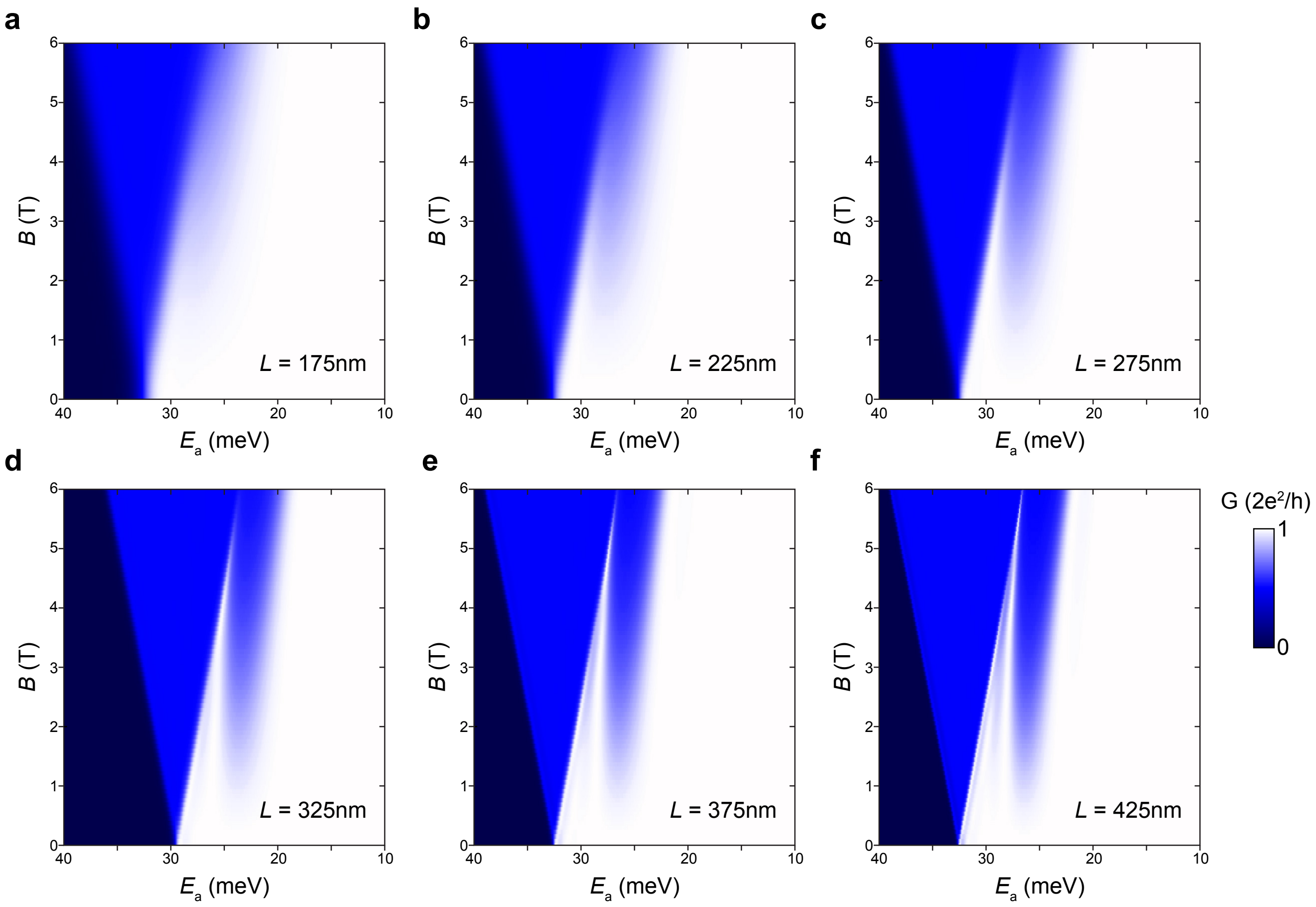

Supplementary Figure 8: Simulations for a QPC of different lengths at fixed angle $\theta = 17°$, $l_{SO} = 20\,$nm. The contact spacing L is increased in steps of 50 nm starting from $L = 175\,$nm (**a**), up to $L = 425\,$nm (**f**). The simulations demonstrate the reduced visibility of the helical gap in short devices. At increasing channel length the conductance dip becomes sharper and sets on at lower magnetic fields. A clear reentrant feature can only be seen in **d, e, f,** which are at the limit of experimental capabilities (Supplementary Fig. 7).

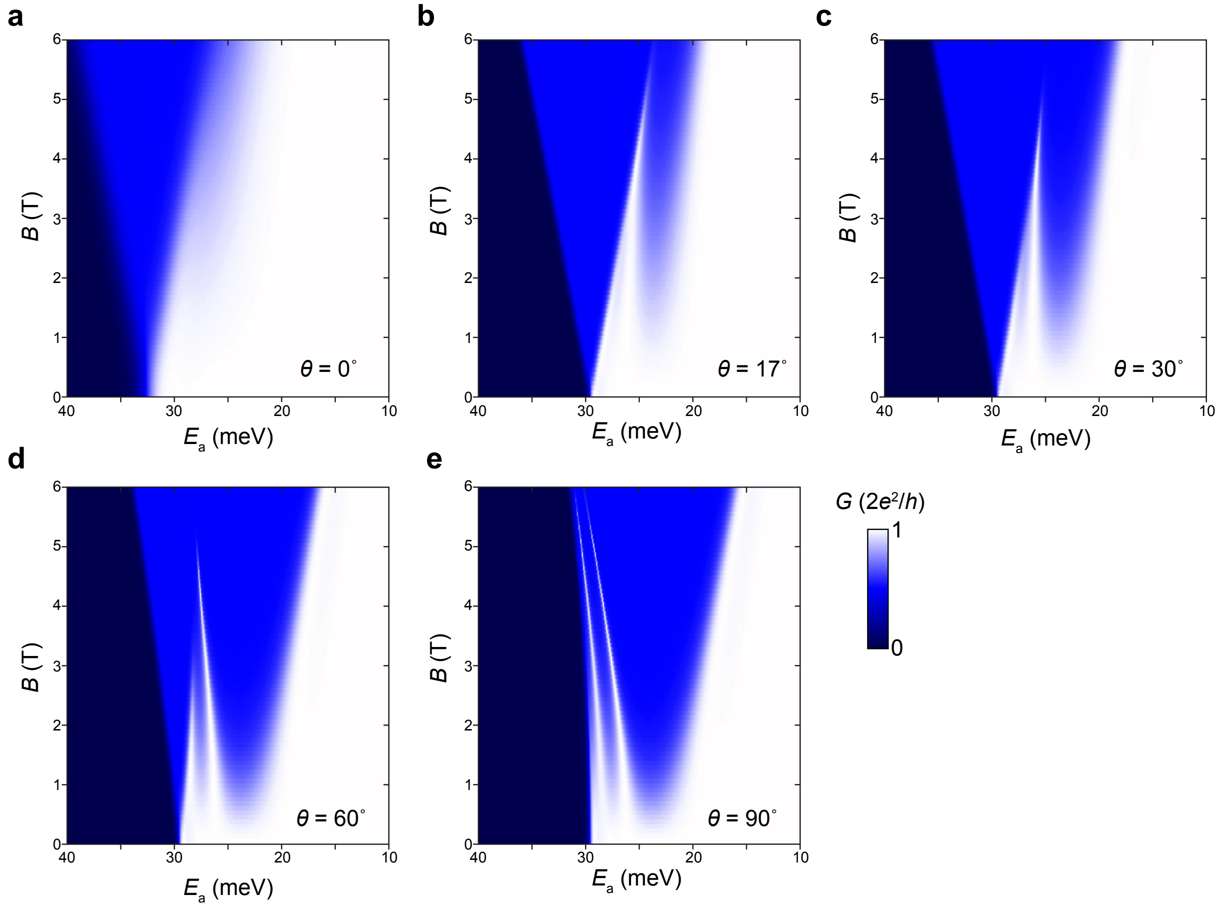

Supplementary Figure 9: Simulations of the angle dependence for a QPC with fixed length $L = 325\,\mathrm{nm}$**.** θ is the angle between $\mathbf{B}_{\mathrm{SO}}$ and the applied magnetic field as defined in the main text. **a,** For $\theta = 0°$, $\mathbf{B}_{\mathrm{ext}} \,\|\mathbf{B}_{\mathrm{SO}}$ and the helical gap disappears. **b-e,** at increasing angles θ the width of the helical gap increases and the width of the inital $0.5 \cdot G_0$ plateau decreases.

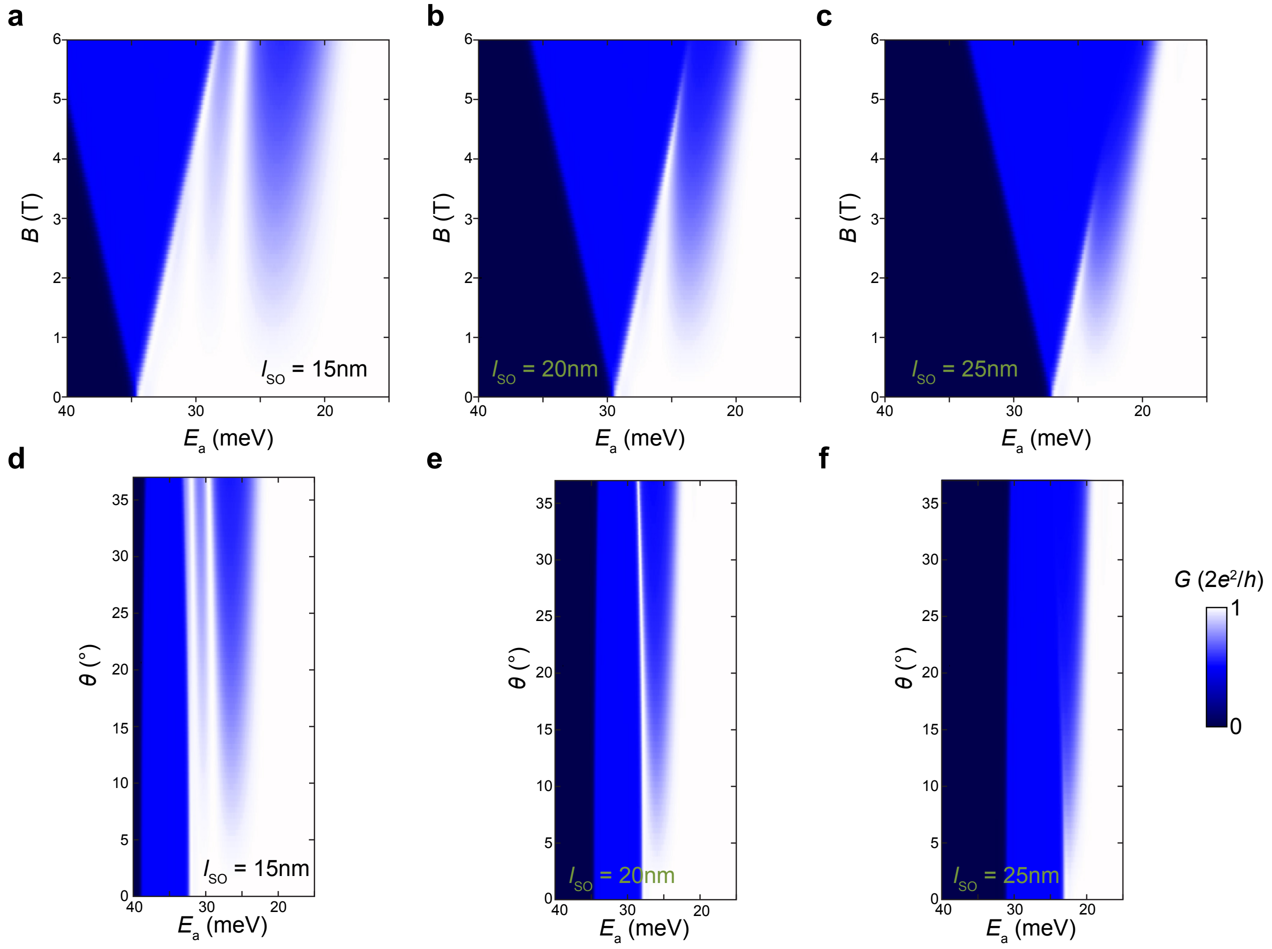

Supplementary Figure 10: Simulations of the magnetoconductance for varying l_{SO}. Variations of $l_{SO} = 1/k_{SO}$ strongly influence the visibility of the helical gap in QPC conductance measurements. The simulations for **a, b, c,** used identical QPC length $L = 325\,\text{nm}$ and offset angle $\theta = 17°$

SUPPLEMENTARY REFERENCES

[1] Rainis, D. & Loss, D. Conductance behavior in nanowires with spin-orbit interaction: A numerical study. *Phys. Rev. B* **90**, 235415 (2014).
[2] March, N. The Thomas-Fermi Approximation in Quantum Mechanics. *Adv. Phys.*, **6(21)**, 1-101 (1957).
[3] Logg, A., Mardal, K.A., Wells, G.N. Automated Solution of Differential Equations by the Finite Element Method. *Springer* (2012).
[4] Eyert, V. *J. Comput. Phys.*, **124**, 271 (1996)
[5] Groth, C.W., Wimmer, M., Akhmerov, A.R. & Waintal, X. Kwant: A software package for quantum transport. *New J. Phys.*, **16**, 063065 (2014).
[6] Pershin, Y. V., Nesteroff, J. A. & Privman, V. Effect of spin-orbit interaction and in-plane magnetic field on the conductance of a quasi-one-dimensional system. *Phys. Rev. B*, **69**, 121306 (2004).
